# Editorial: Immunological aspects and immunotherapy in gynecologic cancers

**DOI:** 10.3389/fimmu.2026.1866404

**Published:** 2026-05-28

**Authors:** Bastian Czogalla, Kanjoormana Aryan Manu, Khayal Gasimli, Anna Jaeger

**Affiliations:** 1 Department of Obstetrics and Gynecology, LMU University Hospital, LMU Munich, Munich, Germany; 2 Department of Immunology, Amala Cancer Research Centre, Thrissur, Kerala, India; 3 Department of Gynaecology and Obstetrics, Johann Wolfgang Goethe University, Frankfurt am Main, Germany; 4 Department of Gynecology, University Medical Center Hamburg-Eppendorf, Hamburg, Germany

**Keywords:** biomarkers, gynecologic cancers, immune checkpoint inhibitors, immune evasion, immunotherapy, personalized medicine, tumor microenvironment

## Introduction

Gynecologic malignancies - including endometrial, ovarian, cervical, vulvar, and vaginal cancers - remain a substantial global health burden. Despite advances in surgery, chemotherapy, and targeted therapies, outcomes for advanced or recurrent disease are still limited. In recent years, immuno-oncology has emerged as a transformative pillar in cancer therapy, with immune checkpoint inhibitors demonstrating clinically meaningful benefit in selected patient populations. However, response rates remain heterogeneous, underscoring the need for a deeper understanding of tumor–immune interactions and the identification of robust predictive biomarkers.

This Research Topic brings together 34 articles spanning basic, translational, and clinical research, offering a comprehensive overview of the immunological landscape of gynecologic cancers and the evolving role of immunotherapy. Collectively, these contributions highlight both the remarkable progress achieved and the significant challenges that remain.

## The immunological landscape and tumor microenvironment

A central theme emerging from this Research Topic is the critical role of the tumor microenvironment (TME) in shaping disease progression and therapeutic response. Multiple studies emphasize the complexity of immune cell infiltration across gynecologic malignancies, revealing both tumor-promoting and tumor-suppressive immune populations (Wang et al.; Yin et al.; Wang et al.; Rothe et al.; Mlambo et al.).

In ovarian and cervical cancers, macrophage polarization appears to play a pivotal role, with M2-like macrophages contributing to immunosuppression and tumor progression, while M1-associated pathways may exert protective effects (Liu et al.; Yin et al.; Wang et al.).

High-resolution approaches such as single-cell RNA sequencing and spatial analyses provide novel insights into the dynamic interplay between tumor cells and immune compartments (Wang et al.). These findings are complemented by studies investigating specific immune cell subsets, including CD8+ T cells and dendritic cells (Rothe et al.; Joo et al.), which further refine our understanding of local immune responses, particularly in mucosal tissues.

Importantly, the prognostic relevance of distinct immune signatures is increasingly recognized. Several contributions demonstrate that specific immune cell populations and their functional states are closely linked to clinical outcomes (Rothe et al.; Zhou et al.; Jiang et al.).

## Mechanisms of immune evasion and resistance

Despite the presence of tumor-infiltrating immune cells, many gynecologic cancers successfully evade immune surveillance. This Research Topic highlights multiple mechanisms contributing to immune escape, including immune checkpoint upregulation, T-cell dysfunction, and immunosuppressive signaling pathways within the TME (Zhang et al.; Pirš et al.; Fu et al.).

Particular attention is given to emerging checkpoint molecules beyond the classical PD-1/PD-L1 axis, such as LAG-3, which are implicated in T-cell exhaustion and may represent promising therapeutic targets (Zhang et al.).

Resistance to immunotherapy remains a major clinical challenge. Reviews within this Topic provide an in-depth overview of resistance pathways in high-grade serous ovarian cancer, illustrating how tumor-intrinsic and microenvironmental factors converge to limit therapeutic efficacy (Fu et al.; Brown et al.).

## Advances in immunotherapy and clinical application

The clinical implementation of immunotherapy in gynecologic cancers is another key focus of this Research Topic. Real-world data and retrospective analyses confirm the activity of immune checkpoint inhibitors across different tumor entities (Kuang et al.; Wang et al.; Gao et al.).

Combination strategies are increasingly explored to enhance therapeutic efficacy. Several studies investigate the integration of checkpoint inhibitors with chemotherapy, demonstrating encouraging activity and manageable safety profiles (Yang et al.; Xiang et al.).

Notably, rechallenge strategies and real-world outcome analyses provide important insights into treatment sequencing and long-term disease control (Yu et al.; Pacholczak-Madej et al.). Individual case reports further illustrate the potential for durable responses even in advanced disease stages (Jin et al.).

Beyond checkpoint inhibition, innovative approaches such as tumor-infiltrating lymphocyte (TIL) therapy and antibody-drug conjugates are discussed, reflecting the rapidly evolving therapeutic landscape (Zhang et al.; Brown et al.; Su et al.).

In addition, health economic considerations are increasingly relevant, as highlighted by cost-effectiveness analyses evaluating novel immunotherapeutic combinations (Xiang et al.).

## Biomarkers and personalized immunotherapy

A recurring theme across multiple contributions is the search for reliable biomarkers to guide patient selection. Microsatellite instability (MSI) and mismatch repair deficiency are further explored across different gynecologic cancers and confirmed as relevant predictors of response (Kuang et al.; Wang et al.).

In addition, novel biomarkers are proposed, including gene expression signatures and immune-related proteins (Liu et al.; Zhou et al.; Pirš et al.). The role of systemic markers such as the neutrophil-to-lymphocyte ratio and the occurrence of immune-related adverse events is also examined (Kao et al.).

Interestingly, real-world data point toward unexpected associations, such as endocrine-related factors influencing immunotherapy efficacy (Pacholczak-Madej et al.), highlighting the complex interplay between host factors and treatment response.

Despite these advances, no single biomarker has yet achieved universal applicability. This underscores the need for integrative approaches combining molecular, immunological, and clinical parameters.

## Clinical challenges and future directions

While the contributions to this Research Topic collectively demonstrate the promise of immunotherapy, they also highlight persistent challenges. Response rates remain limited in several tumor entities, particularly in ovarian cancer, and resistance frequently develops even in initially responsive patients (Mi et al.; Lian et al.).

Future research must focus on refining patient selection, optimizing combination strategies, and identifying novel therapeutic targets. The integration of multi-omics technologies and spatial profiling will be essential to capture the dynamic nature of tumor–immune interactions (Liang et al.; Liu et al.).

Furthermore, rare gynecologic malignancies and underrepresented patient populations require increased attention, as current evidence remains scarce (Zhao et al.; Gao et al.).

Finally, the translation of immunological insights into clinical practice will depend on close collaboration between basic scientists, translational researchers, and clinicians.

## Conclusion

This Research Topic provides a comprehensive and timely overview of the immunological mechanisms underlying gynecologic cancers and the rapidly evolving field of immunotherapy. The collected works underscore the complexity of tumor–immune interactions while highlighting promising avenues for therapeutic innovation. Continued research efforts are essential to overcome current limitations and to translate these advances into improved outcomes for patients with gynecologic malignancies.

